# Public Awareness and Molecular Characterization of *Streptococcus suis* in a High-Incidence Region of Thailand

**DOI:** 10.3390/vetsci13050458

**Published:** 2026-05-08

**Authors:** Perm Premphoolsawat, Khomson Satchasataporn, Thitichai Jarudecha, Kamonwan Lunha, Suganya Yongkiettrakul, Anusak Kerdsin, Daisuke Takamatsu, Nattakan Meekhanon

**Affiliations:** 1 Department of Veterinary Nursing, Faculty of Veterinary Technology, Kasetsart University, Bangkok 10900, Thailand; perm.pre@ku.th (P.P.); cvtkss@ku.ac.th (K.S.); thitichai.j@ku.th (T.J.); 2 National Center for Genetic Engineering and Biotechnology, National Science and Technology Development Agency, Pathum Thani 12120, Thailand; kamonwan.lun@ncr.nstda.or.th (K.L.); suganya.yon@biotec.or.th (S.Y.); 3 Faculty of Public Health, Kasetsart University Chalermphrakiat Sakon Nakhon Province Campus, Sakon Nakhon 47000, Thailand; anusak.ke@ku.th; 4 Department of Bacterial Diseases, National Institute of Animal Health, National Agriculture and Food Research Organization, Tsukuba 305-0856, Ibaraki, Japan; takamatsu.daisuke933@naro.go.jp

**Keywords:** knowledge, attitudes, practices, *Streptococcus suis*, zoonoses

## Abstract

*Streptococcus suis* is a major zoonotic pathogen, with increasing human cases in Nakhon Ratchasima Province, northeastern Thailand. This study aimed to assess residents’ knowledge, attitudes, and practices (KAP) regarding infection risk and to characterize *S. suis* isolates from pigs. A structured questionnaire was administered to 500 residents to evaluate awareness and behaviors related to *S. suis* infection. Nasopharyngeal swabs were collected from slaughtered pigs at three slaughterhouses in Nakhon Ratchasima Province. Presumptive *Streptococcus* isolates were confirmed as *S. suis* by PCR and further characterized by molecular serotyping, virulence-associated gene profiling, and multilocus sequence typing (MLST). Among residents, mean scores suggested generally high levels of KAP; however, misunderstandings regarding transmission persisted. Residents expressed strong readiness to reduce infection risk. Of 285 pig samples, 122 (42.8%) were positive for *S. suis*, representing 16 serotypes. Serotype 9 (10.3%) predominated with several serotypes that have been reported in human infections. The predominant virulence-associated gene profile was *mrp*^−^*/sly^−^/epf*^−^ (77.4%). MLST identified eight STs with five novel STs (ST3147-3151), indicating substantial genetic diversity. Persistent misconceptions despite generally acceptable KAP levels highlight the need for targeted public health education. The high prevalence and genetic variability of *S. suis* in pigs support ongoing surveillance to monitor potentially pathogenic strains.

## Simple Summary

*Streptococcus suis* is a bacterium that can spread from pigs to humans and cause serious illness. In northeastern Thailand, human infections have been increasing, especially in Nakhon Ratchasima Province. This study assessed local residents’ knowledge, attitudes, and practices related to infection risk, and characterized *S. suis* strains in pigs. We surveyed 500 residents and collected nasopharyngeal swabs from pigs at slaughterhouses for microbiological analysis. Most participants had good awareness and were willing to reduce their risk, although misunderstandings about transmission routes remained. *S. suis* was detected in nearly half of the samples and showed high strain diversity, including types previously linked to human infection. These findings support targeted public education and continued surveillance to reduce the risk of human *S. suis* infection.

## 1. Introduction

*Streptococcus suis* is a significant zoonotic pathogen that poses a serious public health concern, especially in regions where close contact with pigs and the consumption of raw or undercooked pork are common. Human infections caused by *S. suis* can lead to serious clinical conditions, including meningitis, septicemia, endocarditis, arthritis, and permanent hearing loss [1,2,3]. Transmission to humans typically occurs through direct contact with infected pigs or contaminated pork products, with the consumption of raw pork being a major risk factor in several Southeast Asian countries, particularly Thailand and Vietnam [4,5,6,7,8].

Thailand currently has the highest reported incidence rate of *S. suis* infection at 0.487 per 100,000 population, followed by Vietnam at 0.249 per 100,000 [9]. The first documented human cases in Thailand were reported in 1987 and involved two cases of meningitis. Since then, multiple outbreaks have been reported across the country, with particularly significant incidents occurring in northern provinces. Notable outbreaks have been reported in several provinces [10,11,12]. In Lamphun in 2000, 10 fatalities were documented. In Phayao, an outbreak in 2007 resulted in 29 confirmed cases and 3 fatalities, followed by another outbreak in 2010 with 25 confirmed cases and 1 fatality. In Phetchabun in 2010, 14 confirmed cases and 5 fatalities were reported.

In recent years, Nakhon Ratchasima Province has emerged as a high-incidence area. From 2019 to 2024, annual confirmed cases ranged from 61 (2.33 per 100,000) to 143 (5.43 per 100,000), with higher incidence in recent years despite year-to-year variation [13]. A localized outbreak occurred in 2021, resulting in 21 confirmed cases [11]. These trends highlight the growing public health burden of *S. suis* infection in the region. Despite ongoing public health interventions, the cultural practice of consuming raw pork continues, and public awareness of foodborne zoonoses remains limited among the general population. Therefore, Nakhon Ratchasima Province represents an important setting for integrated investigation of community risk awareness and the molecular characteristics of local *S. suis* isolates.

Molecular characterization of *S. suis* isolates plays a critical role in understanding the virulence, transmission, and evolution of *S. suis*. Techniques such as serotyping, identification of virulence-associated genes, and multilocus sequence typing (MLST) are commonly used to determine the genetic profiles of both clinical and non-clinical strains. MLST assigns sequence types (STs) based on allelic profiles of seven housekeeping genes (*cpn60*, *dpr*, *recA*, *aroA*, *thrA*, *gki*, and *mutS*) [14], providing insight into the geographical distribution and genetic variation in strains circulating in different regions. MLST data further indicate that human *S. suis* infections cluster within specific lineages. Globally, ST1 is repeatedly reported among human cases, while ST25 and ST28 are more frequently reported among human infections in North America [15]. In Thailand, serotype 2 predominates among human isolates, with ST1 and ST104 reported as major genotypes [11].

Classification of *S. suis* based on capsular polysaccharide antigenicity has identified 29 distinct serotypes [16]. Notably, serotypes 2 and 14 are most frequently implicated in human infections, although other serotypes, including 4, 5, 9, 16, 21, 24, and 31, have also been documented [4,15,17,18,19,20,21]. Moreover, several virulence-associated genes have been characterized, including *epf* (extracellular factor), *mrp* (muramidase-released protein), and *sly* (suilysin) [22,23,24,25]. While the precise functions of EF and MRP remain unclear, they are commonly found in highly virulent strains, particularly those of serotype 2 [26,27]. Notably, suilysin, a hemolysin, exhibits cytotoxic properties and is thought to play a key role in tissue invasion [25,28].

The recent increase in *S. suis* cases in Nakhon Ratchasima Province raises concerns about the zoonotic potential of local strains and insufficient public awareness of food safety, particularly regarding the consumption of raw pork. Therefore, this study aims to analyze the molecular characteristics of *S. suis* isolates in the province. In addition, the study assessed the knowledge, attitudes, and practices (KAP) of local residents via a questionnaire to evaluate the potential risk of *S. suis* infection. Together, these approaches were used to evaluate the potential risk of human *S. suis* infection from a One Health perspective.

## 2. Materials and Methods

### 2.1. Study Design and Setting

**Figure 1:**
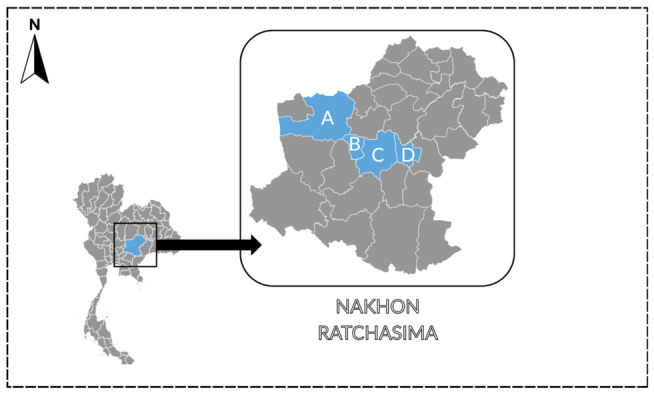
Geographic location of Nakhon Ratchasima Province and the sampling areas. Districts highlighted in blue indicate the sampling locations: A, Dan Khun Thot; B, Kham Thale So; C, Mueang Nakhon Ratchasima; and D, Chaloem Phra Kiat.

This study was conducted as a cross-sectional survey in Nakhon Ratchasima Province, northeastern Thailand, focusing on Chaloem Phra Kiat, Kham Thale So, Dan Khun Thot and Mueang districts, which reported high morbidity rates relative to population size [29]. Dan Khun Thot district, where an *S. suis* outbreak occurred in March 2021, was also included in the survey area. The study locations are shown in Figure 1. The target population consisted of residents living in the selected areas at the time of data collection.

A purposive sampling method was used to select districts with a high incidence of *S. suis* infection, and convenience sampling was applied to interview residents who were accessible and willing to participate during the survey. The sample size was calculated using Yamane’s formula [30].

$$n=\frac{N}{(1+N{\left(e\right)}^{2})}$$

where *n* refers to the required sample size, *N* represents the total population in Nakhon Ratchasima Province (2,635,318 people) [29] and *e* refers to the margin of error (0.05). According to this formula, the required sample size was 400.

### 2.2. Data Collection Tool and Procedures

A structured questionnaire was developed to assess the KAP of participants regarding *S. suis* infection. The questionnaire was designed based on established KAP survey frameworks and published studies on zoonotic and foodborne infections and was adapted to the local study context [31,32,33,34,35]. The questionnaire, written in Thai, comprised four sections: sociodemographic characteristics, knowledge, attitudes, and practices. Before finalization, a pilot test involving 20 respondents was conducted to evaluate item clarity, response patterns, completion time, and the appropriateness and coverage of questionnaire items. Feedback from the pilot was used to refine wording and structure, and no inferential analysis was performed on the pilot data. The finalized questionnaire was then used for the main survey. The sociodemographic section collected information such as gender, age, education level, and monthly income, which were considered potential factors associated with the risk of *S. suis* infection. The knowledge section assessed participants’ background understanding of *S. suis* through ten closed-ended questions with three possible responses (yes, no, and uncertain). To minimize random guessing, only correct responses were scored as 1, while incorrect and uncertain responses were scored as 0. This approach is commonly used in KAP surveys to ensure that scores reflect accurate knowledge rather than chance responses when an uncertain option is provided [31]. Total knowledge scores ranged from 0 to 10 and were categorized as high (8–10), moderate (6–7), or low (0–5). The attitudes section evaluated participants’ concerns about *S. suis* infection, perceived risk, and behavioral tendencies, using five positive statements rated on a five-point agreement scale from 1 (strongly disagree) to 5 (strongly agree). Mean scores of 1–2, 3, and 4–5 indicated negative, neutral, and positive attitudes, respectively. The practices section examined daily behaviors potentially associated with *S. suis* infection, including food preparation, consumption habits, and personal hygiene. It consisted of nine closed-ended questions rated on a five-point frequency scale (1 = never to 5 = always), where mean scores of 1–2, 3, and 4–5 represented high, moderate, and low risk behaviors, respectively.

The participants were recruited through on-site visits with the assistance of Village Health Volunteers (VHVs), who supported the research team in reaching residents in the target area. Residents who were accessible and willing to participate were invited to join the study. This recruitment strategy may have introduced selection bias, as individuals who are more willing to participate in community activities may have been overrepresented. Consequently, the findings may not fully reflect the knowledge, attitudes, and practices of the broader population in the province. However, this approach was considered appropriate for facilitating access to participants within the study setting. Before each interview, participants received a verbal explanation of informed consent, including the study’s objectives, intended use of data, accuracy of responses and voluntary participation. Data were collected through face-to-face interviews using a KAP questionnaire administered by the research team, with logistical support from the VHVs. The data collection was carried out between November 2023 and February 2024.

### 2.3. Data Analysis

Data were analyzed using Stata version 17.0 (Stata Corp LLC, College Station, TX, USA). Descriptive statistics, including frequencies, percentages, median and interquartile range (IQR), were used to summarize sociodemographic characteristics and KAP of participants. Normal distribution was tested by the Shapiro–Wilk test and histogram observation. KAP scores were categorized into groups (e.g., high, moderate, and low levels) prior to statistical analysis. Associations between KAP scores and sociodemographic variables were examined using Pearson’s chi-square (χ^2^) test, with Fisher’s exact test applied when chi-square assumptions were not met. Correlation among knowledge, attitude and practice scores was assessed using Spearman’s rank correlation coefficient (*r_s_*). Statistical significance was set at *p* < 0.05.

### 2.4. Sample Collection and Identification of S. suis

In this study, 285 nasopharyngeal swab samples were collected from slaughtered pigs at three slaughterhouses in Nakhon Ratchasima Province, Thailand, between January 2023 and January 2025. These slaughterhouses receive pigs from multiple farms and therefore provided practical surveillance sites for sampling circulating *S. suis* strains. They were selected based on feasibility and because they represent major pork supply points within the province, consistent with the study objective of characterizing pig-derived *S. suis* in a high-incidence setting and informing potential exposure risk. Each sample was placed in Stuart transport medium (Deltalab, Rubí, Spain) and maintained at 4–8 °C during transport to the laboratory, which occurred within 24 h of collection. Samples were cultured on Todd–Hewitt agar (THA) (Himedia, Thane, India) supplemented with Streptococcus Selective Supplement (Oxoid, Basingstoke, UK) and incubated at 37 °C in 5% CO_2_ for 18–24 h. After incubation, up to six streptococcal-like colonies—small, round, translucent to grayish with smooth margins—were selected from each sample and subcultured on THA under the same conditions for 18–24 h. Up to six presumptive streptococcal colonies were selected per sample to improve recovery of *S. suis* diversity from individual pigs, including less abundant types, while remaining feasible for laboratory processing. To limit overrepresentation of clonal colonies, colonies with the same serotype from the same pig were treated as a single strain and counted once in diversity analyses. All isolates were first screened using the catalase test and then confirmed as *S. suis* by species-specific PCR targeting the *recN* gene [36]. PCR amplification was performed using Taq DNA polymerase (New England Biolabs, Ipswich, MA, USA).

### 2.5. Molecular Characterization of S. suis

The serotypes of *S. suis* isolates were determined using a two-step multiplex PCR based on the method described by Okura et al. [37], targeting capsular polysaccharide synthesis (*cps*) genes. Multiplex PCR reactions were performed using the QIAGEN Multiplex PCR Master Mix (QIAGEN, Hilden, Germany). PCR amplification was performed under standard conditions, including an initial denaturation step, followed by 30 cycles of denaturation, annealing, and extension, and a final extension step. Amplified products were analyzed by agarose gel electrophoresis and interpreted based on expected amplicon sizes. Previously confirmed *S. suis* isolates of known serotypes were used as positive controls. Serotype pairs that cannot be reliably distinguished by the *cps*-based multiplex PCR assay (serotypes 1 vs. 14 and serotypes 2 vs. 1/2) were resolved using the mismatch amplification mutation assay (MAMA)-PCR [38]. MassARRAY was not performed in the present study; however, previously generated MassARRAY-based serotype results were incorporated when available, as described previously [39].

The identification of virulence-associated genes was performed using multiplex PCR targeting *epf*, *sly* and *mrp* genes as described by Silva et al. [40]. The multiplex PCR reactions were performed using the QIAGEN Multiplex PCR Master Mix (QIAGEN, Germany). Samples that tested positive for the *mrp* gene were further analyzed to determine *mrp* variants by PCR using *Taq* DNA polymerase (New England Biolabs, USA). Isolates previously confirmed to carry *epf*, *sly*, or *mrp* were used as positive controls for PCR.

Isolates of *S. suis* were characterized by MLST based on seven conserved housekeeping genes following standard protocols of DNA extraction, PCR amplification, and sequencing. Genomic DNA was extracted using the Omega Bio-tek DNA extraction kit (Omega Bio-tek, Norcross, GA, USA). PCR amplification of the seven housekeeping genes was performed using *Taq* DNA polymerase (New England Biolabs, Ipswich, MA, USA) with primers and cycling conditions described by King et al. [14]. However, amplification of the *mutS* gene failed in some isolates; therefore, primers described by Rehm et al. [41] were used for those samples. PCR products were purified using the NucleoSpin^®^ DNA Purification Kit (Macherey-Nagel, Düren, Germany) and pooled for library preparation prior to sequencing. Amplicons were sequenced using the Illumina platform (paired-end reads, 300 bp). Sequencing reads were quality-filtered and assembled de novo using SPAdes. Allele sequences for each locus were extracted from the assembled contigs and the STs were determined by comparing the obtained allelic profiles with the *S. suis* MLST database (PubMLST; https://pubmlst.org/ssuis/ (accessed on 20 August 2025)). All primers used in this study are listed in Supplementary Table S1.

## 3. Results

### 3.1. Sociodemographic Characteristics of Survey Participants

The sociodemographic characteristics of the respondents are summarized in Table 1. A total of 500 participants residing in the designated study area of Nakhon Ratchasima Province were included in the analysis. The study population was predominantly female (83.4%), with more than half aged ≥ 56 years (51.6%), followed by those aged 46–55 years (30.2%). Most participants reported a monthly income of <15,000 THB (86.0%). Nearly half of the participants had completed primary education (47.0%), while 36.4% had attained secondary education. Regarding food-related practices and awareness, 75.4% of participants reported preparing meals for themselves or their families, and 72.4% indicated having received information on *S. suis* prevention. The majority of participants (88.2%) reported prior awareness of *S. suis* outbreaks.

### 3.2. Knowledge of S. suis Infection and Food Safety

Overall, 64.6% of participants reported having prior knowledge of *S. suis*, with a mean knowledge score of 7.4 ± 1.9 out of 10, corresponding to a moderate level of knowledge. The majority of participants correctly identified pigs as a source of zoonotic transmission (87.0%) and recognized consumption of raw pork as a route of infection (93.2%). However, only 54.6% correctly identified direct contact with raw pork as a potential transmission route, and 45.4% of participants did not recognize direct contact with raw pork as a potential route of *S. suis* transmission, indicating a gap in knowledge regarding transmission.

Regarding knowledge of prevention and clinical features, most participants correctly identified appropriate kitchen hygiene measures, including separation of knives and chopping boards used for raw materials from those used for other foods (78.2%) and wearing gloves when handling raw pork (75.8%). Knowledge of early clinical signs was relatively high, with 78.4% correctly identifying symptoms such as fever, chills, and headache. Most participants recognized the potential severity of infection, with 86.2% acknowledging that *S. suis* infection can be fatal in humans. Nevertheless, 26.8% of participants incorrectly believed that *S. suis* infection could be resolved without seeking medical care. These responses are summarized in Table 2.

### 3.3. Attitudes Toward Risk Behaviors and Prevention

As shown in Table 3, participants reported a neutral level of concern regarding the risk of *S. suis* infection when consuming pork or pork products (median = 3, IQR = 4). In contrast, attitudes were strongly positive toward prioritizing the prevention of *S. suis* infection and recognizing its impact on consumer health (median = 5, IQR = 0 for each statement). Attitudes regarding wound coverage before handling raw pork were generally neutral (median = 4, IQR = 3). Participants also reported a high willingness to change their behavior to reduce the risk of *S. suis* infection (median = 5, IQR = 0).

### 3.4. Food Safety Practices Among Residents

As presented in Table 4, participants reported a moderate frequency of consuming meat or meat products other than pork (median = 3, IQR = 2). High levels of preventive practice were reported for consuming fully cooked pork, washing hands after handling raw pork, and seeking medical care promptly when symptoms occurred following raw pork consumption (median = 5, IQR = 0 for each statement). Participants also reported high agreement that prior information on *S. suis* infection could raise awareness and lead to changes in preventive practices (median = 5, IQR = 0).

In contrast, the use of kitchen gloves when handling raw pork was reported at a low level (median = 1, IQR = 2). Practices related to the separation of chopsticks for raw meat and eating at self-service restaurants were reported at a moderate frequency (median = 3, IQR = 4), whereas using separate chopping boards for raw meat or thorough cleaning of chopping boards before use with other foods was commonly reported (median = 5, IQR = 2). Purchasing pork from certified or standard-approved sources was also frequently reported (median = 5, IQR = 1).

### 3.5. Comparison Between KAP Scores and Sociodemographic Variables

Associations between sociodemographic characteristics and KAP risk levels regarding *S. suis* are presented in Table 5. Knowledge level was significantly associated with gender and educational level (all *p* < 0.05). Higher proportions of good knowledge were observed among female participants, those aged ≥26 years, participants in higher income categories, and those with secondary education or higher compared with their respective reference groups.

Overall, most participants demonstrated positive attitudes toward *S. suis*. Attitude was not significantly associated with any sociodemographic characteristics (all *p* > 0.05). Descriptively, the highest proportion of positive attitudes was observed among participants aged 26–35 years. Among participants aged ≥36 years, positive attitudes were reported by approximately 75–80%, whereas a lower proportion was observed among those aged 18–25 years.

Most participants were classified as having low-risk practices. Practice risk level was significantly associated with gender (*p* < 0.01), with a higher proportion of females reporting low-risk practices compared with males. No significant associations were observed between practice risk level and age, monthly income, or educational level (all *p* > 0.05).

### 3.6. Correlation Between Knowledge, Attitude and Practice

Based on Evans’ classification [42], correlation coefficients were categorized as very weak (0.00–0.19), weak (0.20–0.39), moderate (0.40–0.59), strong (0.60–0.79), and very strong (0.80–1.00). Spearman’s rank correlations indicated weak but statistically significant associations among knowledge, attitude, and practice risk levels (Supplementary Table S2). Knowledge was positively correlated with attitude (*r_s_* = 0.22, *p* < 0.001), whereas practice risk level was negatively correlated with both knowledge (*r_s_* = −0.18, *p* < 0.001) and attitude (*r_s_* = −0.12, *p* = 0.010).

### 3.7. Molecular Characteristics of S. suis Isolates

**Figure 2:**
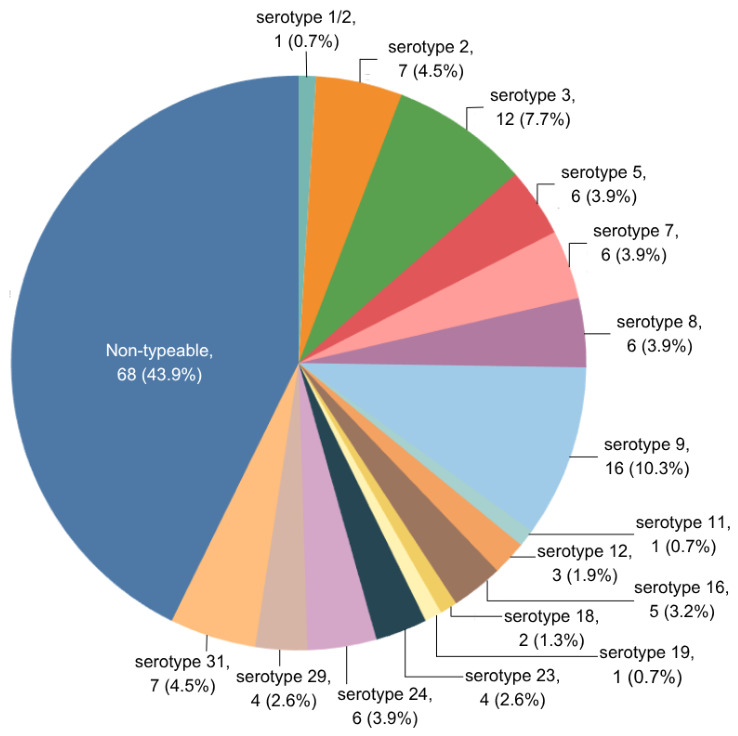
Distribution of serotypes among 155 *S. suis* isolates, *n* (%).

Among the 285 nasopharyngeal swab samples, 122 (42.8%) were confirmed as *S. suis* positive. From these positive samples, a total of 155 *S. suis* isolates were obtained, and multiple isolates were recovered from 28 samples. Serotype identification of the 155 isolates showed that 87 (56.1%) were classified into 16 serotypes, with serotype 9 being predominant (10.3%, n = 16), followed by serotype 3 (7.7%, n = 12) and serotypes 2 and 31 (4.5%, n = 7 each). The remaining 68 isolates (43.9%) were non-typable. The overall serotype distribution is presented in Figure 2.

Based on virulence-associated gene detection, 35 of 155 isolates (22.6%) carried at least one virulence-associated gene (Table 6). The most prevalent virulence-associated gene profile was *mrp*^−^/*sly*^−^/*epf*^−^ (120/155, 77.4%), followed by *mrp*^+^/*sly*^+^/*epf*^−^ (17/155, 11.0%), *mrp*^+^/*sly*^−^/*epf*^−^ (10/155, 6.5%) and *mrp*^−^/*sly*^+^/*epf*^−^ (8/155, 5.2%). Notably, the *epf* gene was not detected in any *S. suis* isolates in this study. Among the 27 isolates positive for the *mrp* gene, four *mrp* variants were identified: *mrp*^s^ (4/27, 14.8%), *mrp*^+^ (7/27, 25.9%), *mrp*^⁎^ (1/27, 3.7%), and *mrp*^⁎⁎⁎^ (1/27, 3.7%). However, fourteen isolates (14/27, 52%) exhibited atypical amplicons of approximately 500 bp or showed no detectable bands that did not correspond to any known variant patterns. These isolates were designated as *mrp*^UT^, indicating divergence within the *mrp* locus that could not be classified by the current PCR scheme.

Among the 87 *S. suis* isolates with assigned serotypes, 13 isolates representing serotypes 1/2, 2, 5, 9, 16, and 24 were selected for MLST analysis. The selected isolates were assigned to eight distinct STs, including ST28, ST2938, ST2942, ST3147, ST3148, ST3149, ST3150, and ST3151. Five STs (ST 3147-ST3151) were novel and have been registered in the *S. suis* PubMLST database. Both ST2942 and ST3147 were single-locus variants of the outbreak-associated ST1688, indicating close allelic relatedness. The distribution of STs among 13 isolates is summarized in Supplementary Tables S3 and S4. The molecular characteristics of individual isolates, including serotypes, virulence-associated gene profiles, and STs, are summarized in Supplementary Table S5.

## 4. Discussion

In addition to causing substantial economic losses to the swine industry, *S. suis* is an important zoonotic pathogen capable of causing severe infections in humans [43]. Thailand is recognized as one of the countries with the highest reported incidence of human *S. suis* infections worldwide [9], with most outbreaks reported in the northern and northeastern regions [11,44]. In recent years, the reported incidence of human *S. suis* infection in Nakhon Ratchasima Province has been relatively high compared with other provinces and has shown an increasing trend [13]. Interestingly, despite the central region having a higher density of swine production [45], human *S. suis* infections are reported less frequently [46]. In this study, residents demonstrated varying levels of KAP related to *S. suis* infection risk, and, in parallel, a relatively high prevalence of *S. suis* was observed among pigs in Nakhon Ratchasima Province.

Assessment of residents’ KAP indicated generally good awareness of *S. suis* infection and overall positive preventive attitudes and behaviors. However, despite relatively high knowledge levels, a substantial proportion of participants held misconceptions about transmission routes, particularly the belief that infection risk is limited to pork consumption. This finding indicates an incomplete understanding of non-dietary exposure pathways, such as direct contact with raw pork during handling or preparation. It should be noted that the KAP findings may not be fully representative of all residents in Nakhon Ratchasima Province, as purposive and convenience sampling methods were used, which may have introduced selection bias toward individuals more accessible and willing to participate.

Attitudes and practices were largely positive, reflecting strong support for preventive measures and a willingness to reduce risky behaviors. Attitude has been recognized as an important determinant of health awareness and preventive action [47], which aligns with the positive relationships observed between knowledge and attitude. However, both knowledge and attitude were weakly and negatively correlated with practices, indicating a gap between awareness and actual behavioral change. Similar risky practices have been reported previously in northern Thailand, where knowledge alone was insufficient to improve practices due to habitual and cultural influences [32], indicating that such practices may be culturally embedded and resistant to change.

Analysis of factors associated with KAP further indicates that sociodemographic characteristics play an important role in shaping awareness and preventive behaviors related to *S. suis* infection. Differences in knowledge were influenced by gender and educational level. Additionally, preventive practices were more strongly associated with gender, with females generally reporting safer behaviors. These findings are consistent with previous studies conducted in Nan and Chiang Mai Provinces, which similarly reported better preventive practices among female participants [32,48].

Overall, the results from the present study suggest that preventive behaviors are influenced by factors beyond individual awareness, including routine food preparation practices and established dietary practices. Previous studies have shown that human *S. suis* infection in Southeast Asia is shaped by multiple interacting factors, such as knowledge, beliefs, gender, age, and socioeconomic conditions [44]. Evidence from Phayao Province further demonstrates that public education initiatives can reduce infection incidence following outbreaks; however, these effects may diminish over time if preventive messages are not consistently reinforced. The recurrence of infections during periods associated with traditional festivals, when raw pork consumption is common, underscores the importance of sustained and culturally appropriate prevention strategies [44].

Compared with previous studies conducted in Thailand, the prevalence observed in the present study is consistent with the wide range reported across different regions and study designs, indicating variability in *S. suis* detection among swine populations. Wongnak et al. [49] reported a lower prevalence of *S. suis*, with 10.9% (54/496) of pig-associated samples and 5.21% (25/480) of environmental samples testing positive in four pork supply chains in Bangkok. In comparison, Meekhanon et al. [45] reported a prevalence of 37% using samples collected from both farms and slaughterhouses in Nakhon Pathom and Ratchaburi Provinces, central Thailand. More recently, Lunha et al. [36] reported a higher prevalence of 60.25% from nasopharyngeal samples collected at slaughterhouses in the central region, whereas Mala et al. [50] reported a markedly lower prevalence of 7.7% from nasopharyngeal samples collected at slaughterhouses in five northern provinces. These differences may be attributable to multiple factors, including geographic region, sample size, study design, sampling period, specimen type, laboratory methods, and variations in swine population density and production systems across study areas.

Serotype analysis in the present study showed that serotypes previously reported in human infections (serotypes 2, 4, 5, 9, 14, 16, 21, 24 and 31) accounted for approximately 30% of all isolates. Among these, serotype 2 is recognized as the most virulent and is strongly associated with severe human infection [51]. When compared with previous studies conducted in the central region of Thailand, Lunha et al. [36] and Meekhanon et al. [45] reported lower proportions of isolates belonging to serotypes previously associated with human infection (21% and 22%, respectively). Although serotype alone does not allow inference of actual zoonotic risk or pathogenicity, the relatively higher proportion of serotypes previously reported in human infections suggests the presence of lineages with possible zoonotic relevance. However, risk assessment should also incorporate virulence-associated gene profiles and MLST backgrounds, as most STs identified in this study differ from those commonly reported among human cases in Thailand, and ST28 is the main lineage previously linked to human isolates [20]. In addition, the proportion of non-typeable isolates observed in this study was comparable to that reported previously in Thailand [36,45,50].

Analysis of virulence-associated genes revealed that the most common gene profile was *epf***^−^/***sly***^−^/***mrp***^−^** (77.4%), which is consistent with a previous report describing a similar predominance of this profile (73%) [45]. These findings indicate that *S. suis* strains lacking all three major virulence-associated genes were predominant among the isolates obtained from nasopharyngeal swabs in this study. However, strains with higher virulence potential could be present at low abundance in the upper respiratory tract and may be missed or under-detected in cross-sectional sampling. Compared with a previous study [45], the proportion of *sly*-positive isolates identified in the present study was lower (16% vs. 27%), whereas the proportion of *mrp*-positive isolates was comparable between studies (17% vs. 18%). Such differences may reflect variations in strain distribution across geographic regions. Notably, the absence of *epf* in both studies suggests that this virulence determinant may play a limited role among *S. suis* strains circulating in slaughtered pigs in Thailand. Furthermore, recent evidence indicates that strains lacking all three of these virulence-associated genes can still cause disease in experimental animals [52], suggesting that pathogenicity may be mediated by additional genetic factors. Accordingly, further investigations targeting other virulence-associated genes are warranted to improve understanding of the pathogenic mechanisms of *S. suis*.

MLST analysis demonstrated considerable ST diversity among *S. suis* isolates from Nakhon Ratchasima Province, including ST28, ST2938, ST2942, and five novel STs (ST3147–ST3151). The detection of ST28 is notable, as this ST has been frequently associated with diseased pigs and has also been reported in human infections [15,53]. ST2942 was the most prevalent ST identified in the present study. To visualize allelic relationships among the identified STs, a goeBURST snapshot was generated using PHYLOViZ Online based on MLST allelic profiles (Supplementary Figure S1). The network showed that the STs detected in this study formed a connected cluster, indicating close allelic relationships among several lineages. The novel STs were positioned within this network and were closely related to previously described porcine lineages. ST3147 was a single-locus variant of the outbreak-associated ST1688, whereas the remaining novel STs (ST3148–ST3151) were located within the same connected goeBURST network as established pig-associated STs. Given the limited number of MLST-typed isolates, these relationships should be interpreted cautiously and confirmed in future studies with larger datasets and complementary phylogenetic analyses.

Importantly, strains isolated from healthy pigs may still exhibit pathogenic potential, as shown for ST1689 within clonal complex 94, which displayed virulence comparable to human-associated ST94 strains in vitro [54]. Therefore, continued molecular surveillance remains important to monitor ongoing genetic diversification of *S. suis* and the potential emergence of pathogenic lineages.

## 5. Conclusions

This study shows that, although residents in Nakhon Ratchasima Province generally demonstrated good levels of knowledge, attitudes, and preventive practices regarding *S. suis*, misconceptions about transmission routes and certain high-risk behaviors persist. In parallel, *S. suis* was common among slaughtered pigs in the province, with considerable diversity in serotypes, STs, and virulence-associated gene profiles, including strains with known zoonotic relevance. Together, these findings suggest that *S. suis* may remain a public health concern in Nakhon Ratchasima Province and in comparable high-incidence settings, and they support prioritizing integrated approaches that link microbiological monitoring in pigs with targeted, behavior-focused risk communication. However, these conclusions should be interpreted considering the study’s limitations, including the cross-sectional design, convenience sampling for the KAP survey, sampling restricted to slaughtered pigs within a defined period, and the absence of longitudinal follow-up.

## Figures and Tables

**Table 1:** Sociodemographic characteristics of participants (N = 500).

Variable	Category	*n*	%
Gender	Male	83	16.6
	Female	417	83.4
Age	18–25	7	1.4
	26–35	29	5.8
	36–45	55	11.0
	46–55	151	30.2
	≥56	258	51.6
Monthly Income, THB *	<15,000	430	86.0
	15,000–30,000	49	9.8
	30,001–50,000	11	2.2
	50,001–70,000	2	0.4
	>70,000	8	1.6
Education	Primary school	235	47.0
	Secondary school	182	36.4
	Vocational certificate	49	9.8
	Bachelor’s degree or higher	34	6.8
Do you prepare meals for yourself or your family?	Yes	377	75.4
	No	51	10.2
	Sometimes	72	14.4
Have you received information about *S. suis* prevention?	Yes	362	72.4
	No	138	27.6
Have you previously heard about *S. suis* outbreaks?	Yes	441	88.2
	No	59	11.8

* 15,000 THB equals 462.93 USD.

**Table 2:** Knowledge questions and correct responses regarding *S. suis* (N = 500).

No.	Statements	Correct Response	*n* (%)
1.	Have you had prior knowledge of *S. suis* infection?	Yes	323 (64.6)
2.	*S. suis* can be transmitted from pigs to humans.	Yes	435 (87.0)
3.	*S. suis* can be transmitted from humans to humans.	No	260 (52.0)
4.	Consumption of raw pork can result in *S. suis* infection.	Yes	466 (93.2)
5.	Direct contact with raw pork can result in *S. suis* infection.	Yes	273 (54.6)
6.	Separating knives and chopping boards used for raw materials from those used for other foods can reduce the risk of infection.	Yes	391 (78.2)
7.	Wearing gloves when handling raw pork can reduce the risk of infection.	Yes	379 (75.8)
8.	Early clinical signs of *S. suis* infection include fever, chills, and headache.	Yes	392 (78.4)
9.	*S. suis* infection can be fatal in humans.	Yes	431 (86.2)
10.	*S. suis* infection can be resolved without seeking medical care.	No	366 (73.2)

Each knowledge statement was scored as 1 for a correct response and 0 for an incorrect response. The total knowledge score ranged from 0 to 10, with a mean score of 7.4 (SD = 1.9).

**Table 3:** Attitude questions and median scores regarding *S. suis* (N = 500).

No.	Statements	Median	IQR
1.	You are concerned about the risk of *S. suis* infection when consuming pork or pork products.	3	4
2.	You believe that consumers should prioritize the prevention of *S. suis* infection.	5	0
3.	You believe that *S. suis* infection has an impact on consumer health.	5	0
4.	You believe that if you have a wound on your hand, it should be covered before handling raw pork.	4	3
5.	You are willing to change your behavior to reduce the risk of *S. suis* infection.	5	0

Each attitude statement was rated on a 5-point scale (1 = strongly disagree to 5 = strongly agree). The interquartile range (IQR) is calculated as the difference between the 75th and 25th percentiles.

**Table 4:** Practice questions and median score related to the risk of *S. suis* infection (N = 500).

No.	Statements	Median	IQR
1.	You regularly consume meat or meat products other than pork.	3	2
2.	You always consume fully cooked pork.	5	0
3.	You always wear kitchen gloves when handling raw pork.	1	2
4.	After handling raw pork, you always wash your hands before engaging in other activities.	5	0
5.	You use a separate chopping board for raw meat or always clean the chopping board thoroughly before using it with other ingredients.	5	2
6.	When dining at self-service restaurants, you always use separate chopsticks for raw meat and for eating.	3	4
7.	You usually purchase pork from certified or standard-approved sources.	5	1
8.	If you know that you have consumed raw pork and experienced symptoms such as fever, headache, vomiting, or diarrhea, you will seek medical care immediately.	5	0
9.	If you have previously received information or advice on preventing *S. suis* infection, you believe that this information helps raise awareness and change your behavior.	5	0

Each practice statement was rated on a 5-point scale (1 = never to 5 = always). The interquartile range (IQR) is calculated as the difference between the 75th and 25th percentiles.

**Table 5:** Associations between sociodemographic characteristics and knowledge, attitudes, and practice risk levels regarding *S. suis* (N = 500).

Variable	Knowledge Level, *n* (%)			Test, *p*-Value	Attitude Level, *n* (%)			Test, *p*-Value	Practice Risk Level, *n* (%)			Test, *p*-Value
	Good	Moderate	Low		Positive	Neutral	Negative		Low Risk	Moderate Risk	High Risk	
**Gender**				χ^2^(*df*) = 36.19(9), *p* < 0.001				χ^2^(*df*) = 2.31(2), *p* = 0.315				Fisher, *p* = 0.001
Male	32 (39%)	24 (29%)	27 (33%)		62 (75%)	20 (24%)	1 (1%)		61 (73%)	21 (25%)	1 (1%)	
Female	254 (61%)	115 (28%)	48 (12%)		326 (78%)	77 (19%)	14 (3%)		371 (89%)	43 (10%)	3 (1%)	
**Age**				Fisher, *p* = 0.165				χ^2^(*df*) = 8.54(8), *p* = 0.382				Fisher, *p* = 0.349
18–25	2 (29%)	4 (57%)	1 (14%)		4 (57%)	2 (29%)	1 (14%)		5 (71%)	2 (29%)	0 (0)	
26–35	18 (62%)	6 (21%)	5 (17%)		24 (83%)	4 (14%)	1 (3%)		28 (97%)	1 (3%)	0 (0)	
36–45	33 (60%)	9 (16%)	13 (24%)		44 (80%)	9 (16%)	2 (4%)		47 (85%)	8 (15%)	0 (0)	
46–55	82 (54%)	49 (33%)	20 (13%)		121 (80%)	24 (16%)	6 (4%)		127 (84%)	21 (14%)	3 (2%)	
≥56	151 (59%)	71 (28%)	36 (14%)		195 (76%)	58 (22%)	5 (2%)		225 (87%)	32 (12%)	1 (1%)	
Income, THB *				Fisher, *p* = 0.152				Fisher, *p* = 0.365				Fisher, *p* = 0.275
<15,000	237 (55%)	129 (30%)	64 (15%)		329 (76%)	86 (20%)	15 (4%)		368 (85%)	59 (14%)	3 (1%)	
15,000–30,000	35 (71%)	7 (14%)	7 (14%)		43 (88%)	6 (12%)	0 (0)		45 (92%)	4 (8%)	0 (0)	
30,000–50,000	7 (64%)	1 (9%)	3 (27%)		10 (91%)	1 (9%)	0 (0)		10 (91%)	0 (0)	1 (9%)	
50,000–70,000	1 (50%)	1 (50%)	0 (0)		1 (50%)	1 (50%)	0 (0)		2 (100%)	0 (0)	0 (0)	
>70,000	6 (75%)	1 (13%)	1 (13%)		5 (63%)	3 (37%)	0 (0)		7 (88%)	1 (12%)	0 (0)	
**Education**				χ^2^(*df*) = 18.13(6), *p* = 0.006				χ^2^(*df*) = 4.39(6), *p* = 0.624				Fisher, *p* = 0.815
Primary school	117 (50%)	85 (36%)	33 (14%)		174 (74%)	53 (23%)	8 (3%)		204 (87%)	30 (12%)	1 (1%)	
Secondary school	118 (65%)	37 (20%)	27 (15%)		149 (82%)	29 (16%)	4 (2%)		155 (85%)	25 (14%)	2 (1%)	
Vocational certificate	28 (57%)	10 (20%)	11 (23%)		37 (76%)	10 (20%)	2 (4%)		42 (86%)	6 (12%)	1 (2%)	
Bachelor’s degree or higher	23 (68%)	7 (21%)	4 (11%)		28 (82%)	5 (15%)	1 (3%)		31 (91%)	3 (9%)	0 (0)	

* 15,000 THB equals to 462.93 USD.

**Table 6:** Distribution of virulence-associated gene profiles by serotype among virulence gene–positive *S. suis* isolates (N = 35).

Virulence-Associated Gene Profile	1/2 (n = 1)	3 (n = 11)	7 (n = 4)	8 (n = 6)	9 (n = 6)	12 (n = 1)	19 (n = 1)	23 (n = 4)	Non-Typeable (n = 1)	Total *n* (%)
***mrp*** **^+^** ***/sly*** **^−^** ***/epf*** **^−^**	**1**	**9**	-	-	-	-	-	-	-	10 (6.5%)
*mrp* ^+^	1	-	-	-	-	-	-	-	-	1 (0.7%)
*mrp* ^***^	-	1	-	-	-	-	-	-	-	1 (0.7%)
*mrp* ^UT^	-	8	-	-	-	-	-	-	-	8 (5.2%)
***mrp*** **^−^** ***/sly*** **^+^** ***/epf*** **^−^**	-	**2**	**1**	**5**	-	-	-	-	-	8 (5.2%)
***mrp*** **^−^** ***/sly*** **^−^** ***/epf*** **^+^**	-	-	-	-	-	-	-	-	-	0 (0.0%)
***mrp*** **^+^** ***/sly*** **^+^** ***/epf*** **^−^**	-	-	**3**	**1**	**6**	**1**	**1**	**4**	**1**	17 (11.0%)
*mrp* ^s^	-	-	3	1	-	-	-	-	-	4 (2.6%)
*mrp* ^+^	-	-	-	-	1	-	1	4	-	6 (3.9%)
*mrp* ^*^	-	-	-	-	-	-	-	-	1	1 (0.7%)
*mrp* ^UT^	-	-	-	-	5	1	-	-	-	6 (3.9%)

*mrp*, *sly*, and *epf* indicate muramidase-released protein, suilysin, and extracellular factor, respectively. *mrp* variants (e.g., *mrp*^s^, *mrp*^+^, *mrp*^⁎^, *mrp*^⁎⁎⁎^) were defined based on PCR amplicon patterns. *mrp*^UT^ indicates atypical amplicons or absence of detectable bands not matching known *mrp* variant patterns.

## Data Availability

The original contributions presented in this study are included in the article/Supplementary material. Further inquiries can be directed to the corresponding author(s).

## Supplementary Materials

The following supporting information can be downloaded at https://www.mdpi.com/article/10.3390/vetsci13050458/s1: Table S1: Primers used in this study. Table S2: Spearman’s correlations among knowledge, attitudes, and practices regarding *Streptococcus suis*. Table S3: Distribution of sequence types by serotype among selected *Streptococcus suis* isolates (N = 13). Table S4: Multilocus sequence typing (MLST) allelic profiles of *S. suis* isolates (N = 13). Table S5: Molecular characterization of *Streptococcus suis* isolates based on *cps*-types, virulence-associated gene profiles, and sequence types (N = 13). Figure S1: goeBURST snapshot of *Streptococcus suis* sequence types (STs) identified in this study, with ST1688 included as a reference.

## Abbreviations

The following abbreviations are used in this manuscript:

IQR	Interquartile Range
KAP	Knowledge, Attitudes and Practices
MLST	Multilocus Sequence Typing
PCR	Polymerase Chain Reaction
ST	Sequence Type
THA	Todd–Hewitt Agar
VHV	Village Health Volunteer

## References

[1] Feng Y., Zhang H., Wu Z., Wang S., Cao M., Hu D., Wang C. (2014). *Streptococcus suis* infection: An emerging/reemerging challenge of bacterial infectious diseases?. Virulence.

[2] Panpaeng C., Kamolvit W., Karaketklang K., Jitmuang A. (2025). Clinical characteristics and trends in the antimicrobial susceptibility profile of *Streptococcus suis* infections in a large tertiary hospital, Thailand, 2007–2023. PLoS Neglected Trop. Dis..

[3] Rayanakorn A., Katip W., Goh B.H., Oberdorfer P., Lee L.H. (2020). A risk scoring system for predicting *Streptococcus suis* hearing loss: A 13-year retrospective cohort study. PLoS ONE.

[4] Dutkiewicz J., Sroka J., Zając V., Wasiński B., Cisak E., Sawczyn A., Kloc A., Wójcik-Fatla A. (2017). *Streptococcus suis*: A re-emerging pathogen associated with occupational exposure to pigs or pork products. Part I—Epidemiology. Ann. Agric. Environ. Med..

[5] Rayanakorn A., Goh B.-H., Lee L.-H., Khan T.M., Saokaew S. (2018). Risk factors for *Streptococcus suis* infection: A systematic review and meta-analysis. Sci. Rep..

[6] Brizuela J., Roodsant T.J., Hasnoe Q., van der Putten B.C.L., Kozakova J., Slotved H.C., van der Linden M., de Beer-Schuurman I.G.A., Sadowy E., Sáez-Nieto J.A. (2024). Molecular Epidemiology of Underreported Emerging Zoonotic Pathogen *Streptococcus suis* in Europe. Emerg. Infect. Dis..

[7] Segura M., Calzas C., Grenier D., Gottschalk M. (2016). Initial steps of the pathogenesis of the infection caused by *Streptococcus suis*: Fighting against nonspecific defenses. FEBS Lett..

[8] Mingkwan W. (2011). Clinical Characteristics and the Predicting Factors of Septic Shock caused by *Streptococcus suis* serotype 2 Infection, Uttaradit Hospital. Uttaradit Hosp. Med. Bull..

[9] Rayanakorn A., Ademi Z., Liew D., Lee L.H. (2021). Burden of disease and productivity impact of *Streptococcus suis* infection in Thailand. PLoS Neglected Trop. Dis..

[10] Fongcom A., Pruksakorn S., Mongkol R., Tharavichitkul P., Yoonim N. (2001). *Streptococcus suis* infection in northern Thailand. J. Med. Assoc. Thail..

[11] Kerdsin A. (2022). Human *Streptococcus suis* Infections in Thailand: Epidemiology, Clinical Features, Genotypes, and Susceptibility. Trop. Med. Infect. Dis..

[12] Thongkamkoon P., Kiatyingangsulee T., Gottschalk M. (2017). Serotypes of *Streptococcus suis* isolated from healthy pigs in Phayao Province, Thailand. BMC Res. Notes.

[13] Ministry of Public Health (2025). 2024 Annual Report, Nakhon Ratchasima, Thailand.

[14] King S.J., Leigh J.A., Heath P.J., Luque I., Tarradas C., Dowson C.G., Whatmore A.M. (2002). Development of a multilocus sequence typing scheme for the pig pathogen *Streptococcus suis*: Identification of virulent clones and potential capsular serotype exchange. J. Clin. Microbiol..

[15] Goyette-Desjardins G., Auger J.P., Xu J., Segura M., Gottschalk M. (2014). *Streptococcus suis*, an important pig pathogen and emerging zoonotic agent-an update on the worldwide distribution based on serotyping and sequence typing. Emerg. Microbes Infect..

[16] Wang K., Wu Z., Yao H., Qiu Y., Lu C. (2017). Identification and Detection of Serotype-Specific Genes: Effective Serotyping of *Streptococcus suis*. Curr. Clin. Microbiol. Rep..

[17] Gottschalk M., Xu J., Calzas C., Segura M. (2010). *Streptococcus suis*: A new emerging or an old neglected zoonotic pathogen?. Future Microbiol..

[18] Callejo R., Prieto M., Salamone F., Auger J.P., Goyette-Desjardins G., Gottschalk M. (2014). Atypical *Streptococcus suis* in man, Argentina, 2013. Emerg. Infect. Dis..

[19] Liang P., Wang M., Gottschalk M., Vela A.I., Estrada A.A., Wang J., Du P., Luo M., Zheng H., Wu Z. (2021). Genomic and pathogenic investigations of *Streptococcus suis* serotype 7 population derived from a human patient and pigs. Emerg. Microbes Infect..

[20] Liu F., Zhang S., Erdeljan M., Zhang Y., Chen Z., Li J., Ding L., Zhang L., Sun W., Yu J. (2025). *Streptococcus suis*: Epidemiology and resistance evolution of an emerging zoonotic bacteria. One Health.

[21] Nghia H.D., Hoa N.T., Linhle D., Campbell J., Diep T.S., Chau N.V., Mai N.T., Hien T.T., Spratt B., Farrar J. (2008). Human case of *Streptococcus suis* serotype 16 infection. Emerg. Infect. Dis..

[22] Blume V., Luque I., Vela A.I., Borge C., Maldonado A., Domínguez L., Tarradas C., Fernández-Garayzábal J.F. (2009). Genetic and virulence-phenotype characterization of serotypes 2 and 9 of *Streptococcus suis* swine isolates. Int. Microbiol..

[23] Li L., Zhang Q., Zhao X., Zhou Y., Sun J., Ren J., Zhou D., Luo Y.B., Hu M., Zhang Y. (2021). Rapid Detection of mrp, epf, and sly Genes by Loop-Mediated Isothermal Amplification in *Streptococcus suis*. Foodborne Pathog. Dis..

[24] Vecht U., Wisselink H.J., Jellema M.L., Smith H.E. (1991). Identification of two proteins associated with virulence of *Streptococcus suis* type 2. Infect. Immun..

[25] Zhang Y., Zong B., Wang X., Zhu Y., Hu L., Li P., Zhang A., Chen H., Liu M., Tan C. (2018). Fisetin Lowers *Streptococcus suis* serotype 2 Pathogenicity in Mice by Inhibiting the Hemolytic Activity of Suilysin. Front. Microbiol..

[26] Segura M., Fittipaldi N., Calzas C., Gottschalk M. (2017). Critical *Streptococcus suis* Virulence Factors: Are They All Really Critical?. Trends Microbiol..

[27] Estrada A.A., Gottschalk M., Rendahl A., Rossow S., Marshall-Lund L., Marthaler D.G., Gebhart C.J. (2021). Proposed virulence-associated genes of *Streptococcus suis* isolates from the United States serve as predictors of pathogenicity. Porc. Health Manag..

[28] Vanier G., Segura M., Friedl P., Lacouture S., Gottschalk M. (2004). Invasion of porcine brain microvascular endothelial cells by *Streptococcus suis* serotype 2. Infect. Immun..

[29] Ministry of Public Health (2023). 2022 Annual Report, Nakhon Ratchasima, Thailand.

[30] Yamane T. (1967). Statistics: An Introductory Analysis.

[31] Andrade C., Menon V., Ameen S., Kumar Praharaj S. (2020). Designing and Conducting Knowledge, Attitude, and Practice Surveys in Psychiatry: Practical Guidance. Indian J. Psychol. Med..

[32] Sutthaluang N., Korwanich K., Korwanich N. (2021). Perception and preventive behaviors for *Streptococcus suis* infection of people in Pua sub-district, Pua district, Nan province. Dis. Control. J..

[33] Al Banna M.H., Disu T.R., Kundu S., Ahinkorah B.O., Brazendale K., Seidu A.A., Okyere J., Rahman N., Mondal S., Matubber B. (2021). Factors associated with food safety knowledge and practices among meat handlers in Bangladesh: A cross-sectional study. Environ. Health Prev. Med..

[34] Soon J.M., Wahab I.R.A., Hamdan R.H., Jamaludin M.H. (2020). Structural equation modelling of food safety knowledge, attitude and practices among consumers in Malaysia. PLoS ONE.

[35] ul Haq N., Hassali M.A., Shafie A.A., Saleem F., Farooqui M., Aljadhey H. (2012). A cross sectional assessment of knowledge, attitude and practice towards Hepatitis B among healthy population of Quetta, Pakistan. BMC Public Health.

[36] Lunha K., Chumpol W., Jiemsup S., Yongkiettrakul S., Li J., Kerdsin A., Takamatsu D., Meekhanon N. (2024). Serotype Distribution and Pathotypic Characteristics of *Streptococcus suis* Isolates from Slaughtered Pigs in a High-Density Pig Farming Area in Thailand. Transbound. Emerg. Dis..

[37] Okura M., Lachance C., Osaki M., Sekizaki T., Maruyama F., Nozawa T., Nakagawa I., Hamada S., Rossignol C., Gottschalk M. (2014). Development of a two-step multiplex PCR assay for typing of capsular polysaccharide synthesis gene clusters of *Streptococcus suis*. J. Clin. Microbiol..

[38] Okuhama-Yoshida E., Nakayama M., Hattori M., Takamatsu D., Okura M. (2023). Improvement of the mismatch amplification mutation assay-PCR for discrimination between *Streptococcus suis* serotypes 2 and 1/2. J. Microbiol. Methods.

[39] Jiemsup S., Lunha K., Chumpol W., Meekhanon N., Kerdsin A., Yongkiettrakul S. (2025). Development of a high-throughput MassARRAY-based single assay for the characterization of *Streptococcus suis* species and serotypes. Sci. Rep..

[40] Silva L.M., Baums C.G., Rehm T., Wisselink H.J., Goethe R., Valentin-Weigand P. (2006). Virulence-associated gene profiling of *Streptococcus suis* isolates by PCR. Vet. Microbiol..

[41] Rehm T., Baums C.G., Strommenger B., Beyerbach M., Valentin-Weigand P., Goethe R. (2007). Amplified fragment length polymorphism of *Streptococcus suis* strains correlates with their profile of virulence-associated genes and clinical background. J. Med. Microbiol..

[42] Evans J.D. (1997). Straightforward Statistics for the Behavioral Science.

[43] Staats J.J., Feder I., Okwumabua O., Chengappa M.M. (1997). *Streptococcus suis*: Past and present. Vet. Res. Commun..

[44] Kerdsin A., Segura M., Fittipaldi N., Gottschalk M. (2022). Sociocultural Factors Influencing Human *Streptococcus suis* Disease in Southeast Asia. Foods.

[45] Meekhanon N., Kaewmongkol S., Phimpraphai W., Okura M., Osaki M., Sekizaki T., Takamatsu D. (2017). Potentially hazardous *Streptococcus suis* strains latent in asymptomatic pigs in a major swine production area of Thailand. J. Med. Microbiol..

[46] Jitpeera C., Kripattanapong S., Klaytong P., Rangsiwutisak C., Wannapinij P., Doungngern P., Pinyopornpanish P., Chamawan P., Srisuphan V., Tuamsuwan K. (2025). Epidemiology of *Burkholderia pseudomallei*, *Streptococcus suis*, *Salmonella* spp., *Shigella* spp. and *Vibrio* spp. infections in 111 hospitals in Thailand, 2022. PLoS Glob. Public Health.

[47] Ma X., Bo L., Zhou X. (2024). Knowledge, attitude, and practice toward foodborne disease among Chinese college students: A cross-sectional survey. Front. Public Health.

[48] Pechkul K., Na Lampang K. (2020). Factors Related to Prevention Practice *Streptococcus suis* Infection of People in Chiang Mai Province. Lanna Public Health J..

[49] Wongnak P., Wiratsudakul A., Nuanualsuwan S. (2020). A risk assessment of pathogenic *Streptococcus suis* in pork supply chains and markets in Thailand. Food Control.

[50] Mala W., Prathan R., Hein S., Angkittitrakul S., Bitrus A.A. (2025). Prevalence, antimicrobial resistance characteristics and virulence genes of *Streptococcus suis* in pigs in upper northern Thailand. Thai J. Vet. Med..

[51] Tang J., Wang C., Feng Y., Yang W., Song H., Chen Z., Yu H., Pan X., Zhou X., Wang H. (2006). Streptococcal toxic shock syndrome caused by *Streptococcus suis* serotype 2. PLoS Med..

[52] Li Y., Ma B., Jia X., Wan Y., Ni S., Chen G., Zong X., Jin H., Li J., Tan C. (2025). Population Genomics, Virulence Traits, and Antimicrobial Resistance of *Streptococcus suis* Isolated in China. Microorganisms.

[53] Estrada A.A., Gottschalk M., Rossow S., Rendahl A., Gebhart C., Marthaler D.G. (2019). Serotype and Genotype (Multilocus Sequence Type) of *Streptococcus suis* Isolates from the United States Serve as Predictors of Pathotype. J. Clin. Microbiol..

[54] Hatrongjit R., Boueroy P., Jenjaroenpun P., Wongsurawat T., Meekhanon N., Chopjitt P., Zheng H., Fittipaldi N., Chareonsudjai S., Segura M. (2023). Genomic characterization and virulence of *Streptococcus suis* serotype 4 clonal complex 94 recovered from human and swine samples. PLoS ONE.

