# Supplementary material for: Public Awareness and Molecular Characterization of Streptococcus suis in a High-Incidence Region of Thailand
Source: Vet Sci. 2026 May 8;13(5):458. doi: 10.3390/vetsci13050458 (PMC13211474; doi:10.3390/vetsci13050458)
Supplement: Supplementary file 1 [file vetsci-13-00458-s001.zip › vetsci-4280989-supplementary.pdf]

Supplementary Table S1. Primers used in this study.

Target gene/ Assay	Primer sequence (5´-3´)	Product size (bp)	Reference
recN	F: TTATCTGTCTTGAAACAGATTGGG<br>R: TCTTTCTCTAAGTTCTTAAGCTGAAC	731	[36]
***cps* group I**	F: TGGTTCAAATATCAATGCTC<br>R: ATTGGTTGTGAGTGCATTG	933	[37]
***cps* type 3**	F: GGTTTTGATTGGTCTAGTTG<br>R: CTCTAAAGCTCGATATCTAC	214	[37]
***cps* type 13**	F: TATGGTTAAAGGTGGAACTG<br>R: CCTTGTATATATTCCCTCCA	408	[37]
***cps* type 18**	F: TAATGGGATAGTTGCGTTAC<br>R: ATACATAAAGTTGTCCTGCG	617	[37]
***cps* group II**	F: TCAAAATACGCACCTAAGGC<br>R: CACTCACCTGCCCCAAGAC	823	[37]
***cps* type 2 and 1/2**	F: TTAGCAACGTTGCCAATAAG<br>R: AATCCTCCATTAAAACCCTG	173	[37]
***cps* type 6**	F: GCTCACTATTTTTACATTACAC<br>R: TATTACTCCGCCAAATACAG	278	[37]
***cps* type 1 and 14**	F: TTAGACAGACACCTTATAGG<br>R: CTAGCTTCGTTACTTGATTC	386	[37]
***cps* type 16**	F: AAGGTTATCCACGAAAGATG<br>R: TCCGGCAATATTCTTTCAAG	494	[37]
***cps* type 27**	F: AGACACTGCTTGCATTATTG<br>R: TCAGAATTACTTCCTGTTGC	655	[37]
***cps* group III**	F: TGATTTGGGTGAGACCATG<br>R: CTCATGCTGGATAACACGT	583	[37]
***cps* type 21**	F: TATCATATTGAGAATCTTCCC<br>R: TTGCGTAGCATACAAAGTTC	160	[37]
***cps* type 28**	F: ATTATGTTGGTTGCAGAAGG<br>R: CGACTCAATTGTTGTAGTAG	272	[37]
***cps* type 29**	F: TTCTGGGATTTTAGGAATGC<br>R: CATGAAATACGCACTTGTAC	415	[37]
***cps* type 30**	F: TATTGCACTAGCTTCAGAAC<br>R: TGCATCCATAGTTGTATTCG	568	[37]
***cps* group IV**	F: ACAGTCGGTCAAGATAATCG<br>R: TCAGCTTGGGTAATATCTGG	455	[37]
***cps* type 4**	F: GACTATCTGTATACCCAAAC<br>R: TCCTTCCAAGTATTCTCTAG	903	[37]
***cps* type 5**	F: ATCTTAGGAATGATTCGGAC<br>R: ACCAGATATCTGAGCAAATG	720	[37]
***cps* type 7**	F: AACTACCTACCTGAACTTTG<br>R: AGTCTAAAAGTGATCGAGTC	566	[37]

***cps* type 17**	F: TAGCATCAGTTTATACGAGG<br>R: TAGTTTATCTGTGACACACC	455	[37]
***cps* type 19**	F: GTGTCGCAAATCAAGTATTG<br>R: AAGCTAGTACAACAAGCATG	348	[37]
***cps* type 23**	F: TAATGTATGCTCTGTCACTG<br>R: AACGAAACGGAATAGTTTGC	221	[37]
***cps* group V**	F: GGAAAGATGGAGGACCAGC<br>R: CCAACCAGACTCATATCCCC	265	[37]
***cps* type 8**	F: AAATAAGGTAGGAGCTACTC<br>R: ATCCAACCTTAGCTTTCTGT	446	[37]
***cps* type 15**	F: ATCGTTTTGAGATTGAGTGG<br>R: TAAACGGATTCGGTTACTCA	542	[37]
***cps* type 20**	F: TGTGGATTTCTGGGATAATC<br>R: TGTGGACGAATTACTACTTG	698	[37]
***cps* type 22**	F: GCATTATCAGGATTCTTTCC<br>R: CCAATTGGGTGTTCAAAAAG	296	[37]
***cps* type 25**	F: GTTTGCTCCGATCATAATAG<br>R: CCAGTAAAAGGACTCAATAC	174	[37]
***cps* group III and VI**	F1: GATGCCCCAAGCGATATGCC<br>R1: GGACCAACAATGGCCATCTC<br>F2: GACGCACCAAGTGATATGCC<br>R2: GGTCCGACAATAGCCATTTC	146	[37]
***cps* type 9**	F: GAAAGTAGGTATATCTCAGC<br>R: GGGCTATTAAAACTCCTATC	368	[37]
***cps* type 10**	F: TTTCCCATTTGCTTATGGAC<br>R: GGAATAAAAACGATTGGGAG	633	[37]
***cps* type 11**	F: ATGCGATTGCAACAATTGAC<br>R: AGGCATGAGTAATACATAGG	833	[37]
***cps* type 12**	F: AACAGGTATTTCAGGATTGC<br>R: CTCGGATAAAGATAATCAGC	131	[37]
***cps* type 24**	F: TACTGAGATTTATTGGGACG<br>R: AAGCGATTGGATTACATTGC	224	[37]
***cps* type 26**	F: TTATACCGAAATTTTGTTGCC<br>R: CGTCAATCATATAAAGTGGG	472	[37]
***cps* type 33**	F: GATGTTTTCAACAGGTGTAC<br>R: CAAAGTACCTATTTTCAGCG	710	[37]
***cps* type 31**	F: ACAATCGTTTCTGCAATACG<br>R: GATGAAAACATCGTTGGTAG<br>F: ATCAGTAGTGGGAATAGTTG<br>R: TTTACTGTTTTTCGACCGTG	842<br><br>423	[37]
***cps* type 32**	F: AACCGCTGTTGAATTAAGAG<br>R: TTCGTTAGTTGAACTGTTCC<br>F: TAGGACTATGGTTCCTAATG<br>R: TATTCTAGTTCAAGTCGCTC	570<br><br>342	[37]
***cps* type 34**	F: AAGTTTCATTCGAGGACTTC<br>R: GTATATAACACCGCAAGAAG	246	[37]

	F: ATACAGTGATGTCTTGCAAC<br>R: ATTGCTTTTTGACAATCGGC	701	
**MAMA-PCR**	F1: ATCGCTTTGTGGTGGCCTTY<br>F2: ATCGCTTTGTGGTGGCCTTG<br>R1-2: AGAAGCTTCTTTTGCTGTTTGC	367	[38]
epf	F: CGCAGACAACGAAAGATTGA<br>R: AAGAATGTCTTTGGCGATGG	744	[40]
sly	F: GCTTGACTTACGAGCCACAA<br>R: CCGCGCAATACTGATAAGC	248	[40]
mrp	F: ATTGCTCCACAAGAGGATGG<br>R: TGAGCTTTACCTGAAGCGGT	188	[40]
***mrp* variant**	F: GACAGATGGTGAGGAAAATGG<br>R: TGAGCTTTACCTGAAGCGGT	1148	[40]
dpr	F: CGTCTTTCAGCCCGCGTCCA<br>R: GACCAAGTTCTGCCTGCAGC	~500	[14]
thrA	F: GATTCAGAACGTCGCTTTGT<br>R: AAGTTTTCATAGAGGTCAGC	~500	[14]
cpn60	F: TTGAAAAAACGTRACKGCAGGTGC<br>R: ACGTTGAAIGTACCACGAATC	~500	[14]
recA	F: TATGATGAGTCAGGCCATG<br>R: CGCTTAGCATTTTCAGAACC	~500	[14]
gki	F: GGAGCCTATAACCTCAACTGG<br>R: AAGAACGATGTAGGCAGGATT	~500	[14]
aroA	F: TTCCATGTGCTTGAGTCGCTA<br>R: ACGTGACCTACCTCCGTTGAC	~500	[14]
mutS	F: AAGCAGGCAGTCGGCGTGGT<br>R: AGTACAAACTACCATGCTTC	~500	[41]

Supplementary Table S2. Spearman's correlations among knowledge, attitudes, and practices regarding *S. suis*.

Variable pair	Knowledge	Attitude	Practice
**Knowledge**	1.00		
**Attitude**	0.22[a]	1.00	
**Practice**	-0.18[a]	-0.12[b]	1.00

Superscripts a and b indicate Spearman's rank correlation coefficients (r_s). $p < 0.001$ for correlations marked a, and $p = 0.010$ for correlations marked b.

Supplementary Table S3. Distribution of sequence types by serotype among selected *S. suis* isolates (N = 13).

**Serotype**	**Sequence type (ST) (*n*)**							
	28	2938	2942	3147	3148	3149	3150	3151
1/2	1	0	0	0	0	0	0	0
2	0	0	0	1	0	0	0	0
5	0	0	1	0	0	0	0	0
9	0	1	4	0	0	0	1	0
16	0	0	0	0	0	0	0	1
24	0	0	0	0	2	1	0	0
**Total**	1	1	5	1	2	1	1	1

Supplementary Table S4. Multilocus sequence typing (MLST) allelic profiles of *S. suis* isolates (N = 13).

**Isolate ID**	**Serotype**	**Allelic profile**							**ST**
		aroA	*cpn60*	*dpr*	*gki*	*mutS*	*recA*	*thrA*	
PP5	9	5	485	42	7	14	109	349	2942
PP18	5	5	485	42	7	14	109	349	2942
PP24	2	25	485	545	7	14	109	349	3147
PP32	9	5	485	42	7	14	109	349	2942
PP35	9	5	485	42	7	14	109	349	2942
PP55	24	618	77	23	235	145	265	113	3148
PP67	24	251	145	23	168	27	149	12	3149
PP69	1/2	2	30	5	7	14	109	349	28
PP77	9	5	485	42	7	14	109	349	2942
PP79	9	5	485	5	7	14	109	349	3150
PP88	24	618	77	23	235	145	265	113	3148
PP94	16	23	48	42	31	14	109	12	3151
PP112	9	88	485	30	8	98	15	55	2938

Supplementary Table S5. Molecular characterization of *Streptococcus suis* isolates based on *cps*-types, virulence-associated gene profiles, and sequence types (N=13).

**Isolate ID**	**Serotype**	**Virulence-associated gene profile**			**ST**
		mrp	*epf*	*sly*	
PP5	9	-	-	-	2942
PP18	5	-	-	-	2942
PP24	2	-	-	-	3147
PP32	9	-	-	-	2942
PP35	9	-	-	-	2942
PP55	24	-	-	-	3148
PP67	24	-	-	-	3149
PP69	1/2	+	-	-	28
PP77	9	-	-	-	2942
PP79	9	-	-	-	3150
PP88	24	-	-	-	3148
PP94	16	-	-	-	3151
PP112	9	-	-	-	2938

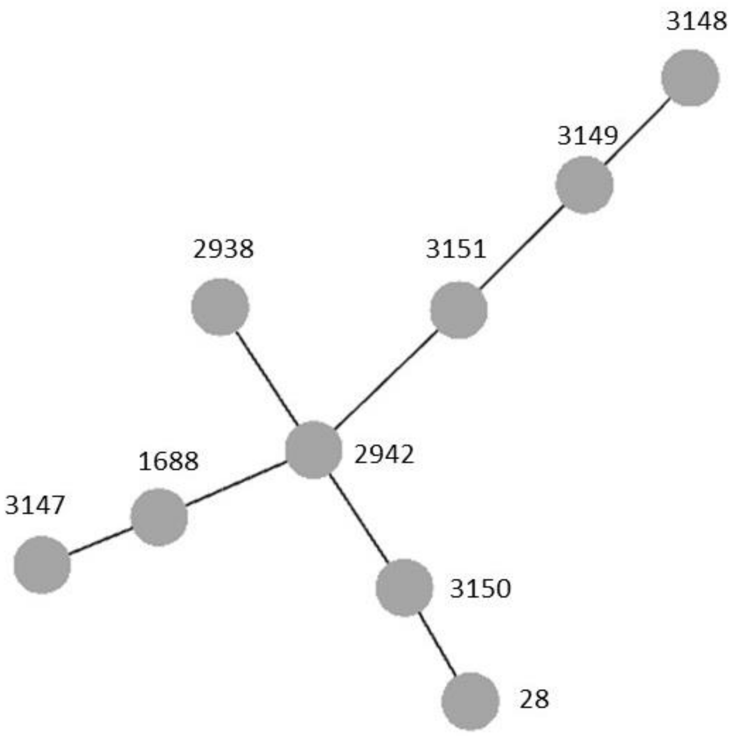

Supplementary Figure S1. goeBURST snapshot of *Streptococcus suis* sequence types (STs) identified in this study, with ST1688 included as a reference. Allelic relationships among STs were visualized using the goeBURST algorithm based on the seven-locus MLST scheme. Nodes represent STs and connecting lines indicate allelic relatedness inferred by goeBURST. ST1688 was included for comparison and was not detected among isolates in the present study.
